# Utilizing Colpocleisis to Repair a Vesicovaginal Fistula in a Cervical Cancer Patient with History of Pelvic Radiation: A Case Report and Literature Review

**DOI:** 10.1155/2021/8865146

**Published:** 2021-05-05

**Authors:** Ahmad Dahman, Daniel McClelland, Stanley Zaslau, Valerie Galvan Turner, Omar Duenas, Robert Shapiro

**Affiliations:** ^1^West Virginia University School of Medicine, Morgantown, WV 26506, USA; ^2^Department of Urology, West Virginia University School of Medicine, Morgantown, WV 26506, USA; ^3^Department of Obstetrics & Gynecology, West Virginia University School of Medicine, Morgantown, WV 26506, USA

## Abstract

**Background:**

Vesicovaginal fistula is a rare and distressing urological condition. It is especially prevalent in developing countries with the predominant etiology secondary to obstructed labor. Radiation therapy in female patients with cervical cancer is a risk factor for vesicovaginal fistula formation in the United States. *Case Presentation*. A 53-year-old woman with a history of cervical cancer and radiation presented with continuous urinary incontinence. Following diagnostic vaginoscopy, a 1 cm vesicovaginal fistula was diagnosed at the vaginal apex. The patient elected for surgical repair. She subsequently underwent successful transvaginal fistula closure using colpocleisis to optimally address the systemic factors of poor wound healing associated with irradiated tissue. Because of the adjacent tissue having been compromised by pelvic radiation, we opted to use a biologic graft made of human cadaveric pericardial tissue (CPT) instead of a native tissue flap to provide additional support for the fistula repair.

**Conclusion:**

A transvaginal approach for surgical repair of vesicovaginal fistula can be successful in patients with a prior history of pelvic radiation. Transvaginal colpocleisis is a viable option to augment vesicovaginal fistula repair for patients with significant comorbidities when sexual intercourse is no longer desired.

## 1. Introduction

Vesicovaginal fistula (VVF) is a distressing gynecologic complication that occurs when an abnormal connection forms between the bladder and the vagina [1]. While rare in the United States, it is still a prevalent issue for women in developing countries with studies projecting upwards of three million women living with unrepaired VVFs [1]. The most common etiology of VVF is obstructed labor, accounting for around 90% of fistulas in women of developing countries [1]. Other risk factors include adjacent malignancy, radiation, and repetitive pelvic operations. Classically, a patient presents with continuous urine leakage from her vagina. If radiation is a contributing factor, this presentation may be delayed by years. Diagnosis is clinical with a relevant history and physical examination but can be confirmed with instillation of methylene blue into the bladder and further characterized with specific imaging studies.

The mainstay of treatment is surgery with the option to approach vaginally or abdominally [2]. The vaginal approach minimizes blood loss, hospital stay, and pain [2]. In the following, we report a case of a 54-year-old woman with a challenging VVF resulting from stage four cervical cancer and history of brachytherapy to the vagina. The fistula was successfully treated with transvaginal colpocleisis.

## 2. Case Presentation

**Figure 1:**
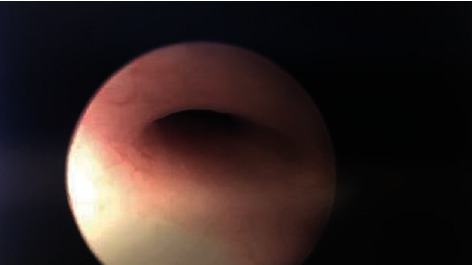
Fistula tract visualized by rigid cystourethroscopy in the urinary bladder.

A 53-year-old woman first presented to our clinic as a referral for evaluation of urinary incontinence. She had a complex history, including stage four cervical cancer nine years prior that had invaded the bladder and been treated with combination brachytherapy, external beam radiation, and chemotherapy. Her urinary incontinence complaint traced back seven months and was characterized as a complete loss of bladder control. Speculum examination revealed the pooling of urine in the vaginal vault with a high suspicion for VVF. The patient did not have evidence of recurrent cervical cancer. Two weeks later, she underwent rigid cystourethroscopy with bilateral ureteral catheterization, bilateral retrograde pyelography, and vaginoscopy. The procedure showed a fistula tract on the anterior vaginal wall extending medially into the bladder just posterior to the trigone (Figure 1). No evidence of upper tract obstruction was seen on retrograde pyelography. No vaginal stricture was present preventing access to the fistula. The fistula tract was biopsied and revealed no evidence for recurrence of her cervix cancer.

**Figure 2:**
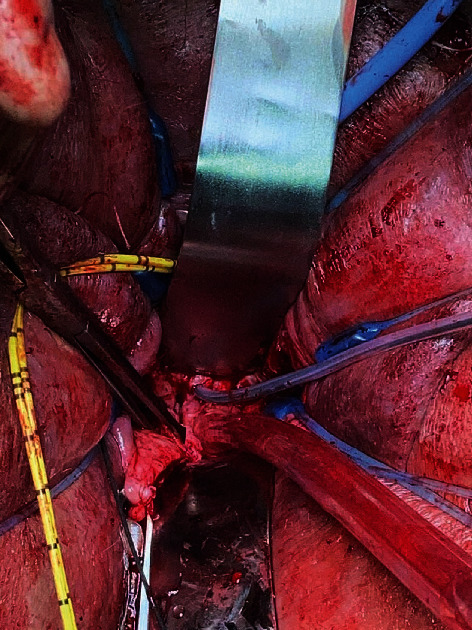
Ureteral catheters (yellow) retracted away from the operative field. A 10F Foley catheter was shown exiting the fistula tract along the anterior vaginal wall just inferior to the metal retractor.

Due to her prior history of pelvic radiation and morbid obesity with a BMI of 54.95 kg/m^2^, a vaginal approach was opted for to limit postoperative complications associated with poor wound healing. We began by inserting a 22 French rigid cystoscope with a 30-degree lens into the bladder through the urethra. Bilateral ureteral orifices were in normal position, and the large fistula tract was noted to be supratrigonal, away from the ureteral orifices. We then placed a 0.035 straight sensor wire into the left ureteral orifice up to the kidney. A 5-French Pollack catheter was passed over this wire and the procedure was repeated on the right side, providing identification of the ureters for the remainder of the procedure. Vaginoscopy was then performed, and a 0.035 straight sensor wire was passed through the fistula opening. We returned to the bladder with the cystoscope, where we grasped the distal end of the sensor wire. A 10-French silicone catheter was placed over this using a modified Seldinger technique with 10 mL in the balloon to identify the fistula tract. The setup was completed and we moved forward with the fistula repair (Figure 2).

**Figure 3:**
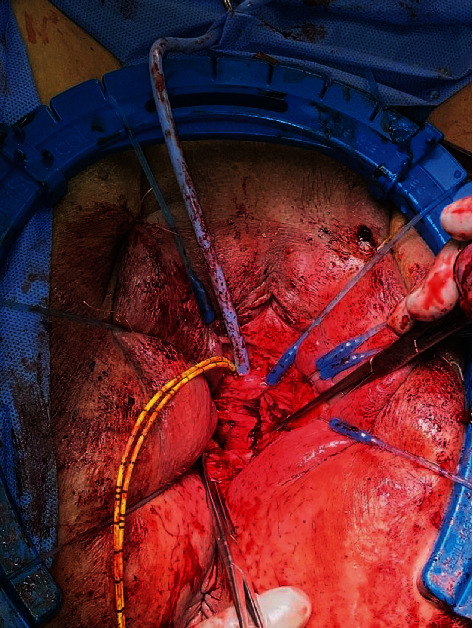
Biologic graft made of human cadaveric dermis placed over the fistula tract using allis clamps.

**Figure 4:**
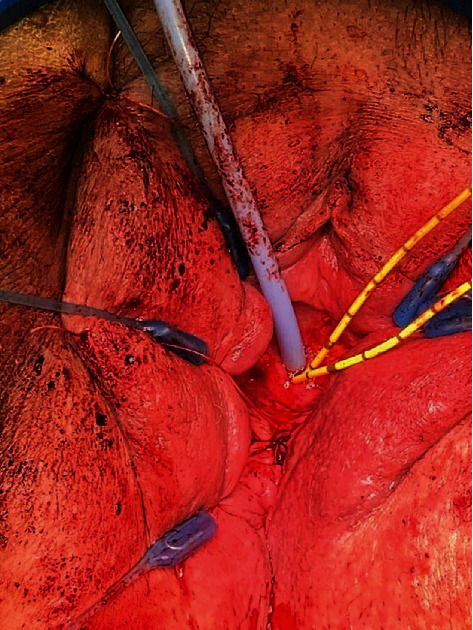
Completed fistula repair using colpocleisis to obliterate the vaginal opening.

We began by placing 3-0 Vicryl sutures on the superior and inferior aspects of the labia majora to provide more retraction. A lone star retractor was placed with skin hooks deeper in the vagina to maximize exposure. Following exposure, vasopressin solution was injected circumferentially around the fistula and posterior vaginal wall to provide hemostasis and hydrodissection. Using a 15-blade scalpel, the anterior vagina was incised circumferentially around the fistulous tract. In the same plane, the vagina was dissected away from surrounding tissue with Metzenbaum scissors. This maneuver was repeated on the posterior aspect of the vagina. After the vaginal flaps were elevated sufficiently, we continued dissection proximally to the fistula. Once around the tract, we used 3-0 Vicryl sutures on a UR6 needle to close the mucosal edges over the fistula tract. A moderate fill test of the bladder revealed no leak. A biologic graft made of CPT was prepped with a soak time of 30 minutes and cut into 2 × 2 cm squares. With the 3-0 Vicryl on a UR6, we placed the dermis in a quilted fashion overlying the repair (Figure 3). Once properly placed, we began sewing the rectovaginal fascia to the pubovesical fascia with 3-0 Vicryl on a UR6 in a tension-free fashion to obliterate the vaginal opening. We concluded by trimming and closing the vaginal mucosa, thereby finishing the colpocleisis to provide the extra layer of support (Figure 4). A final moderate fill test was performed, which ensured no leak.

The patient presented two weeks postoperatively with a negative cystogram. The patient was seen at six months from surgery and continues to be completely dry.

## 3. Discussion

Surgical management of vesicovaginal fistulas includes multiple approaches either abdominally or vaginally. The specific characteristics of each patient help direct the optimal approach. In the case of a patient with significant radiation history, such as ours, access to the vagina may be limited on account of stricture formation. The reported incidence of radiation-induced vaginal stricture among patients with cervical cancer is highly variable ranging from 1% to 88% [3]. This is likely on account of the wide variation in patient, tumor, and treatment factors. In our case, no vaginal stricture was present, and the fistula was easily accessible through the vagina. The vaginal approach may be advantageous over the abdominal approach for wound healing and recovery time, especially amongst patients that are morbidly obese.

Although little research exists regarding the physiologic mechanisms of vaginal wound healing, there are some known observations that may potentially make this an optimal approach for healing compared to the abdominal route. First, the vagina is highly vascularized [4], and because the wound-healing process is mediated by inflammation and neovascularization [5], this feature may account for improved wound healing compared to the abdominal route. Certain growth factors, such as platelet-derived growth factor [6], transforming growth factor [7], and fibroblast growth factor [8], have been shown to play a role in the wound-healing process. In theory, greater concentrations of these factors in the vagina versus the abdomen may improve wound healing.

In a traditional fistula repair, a flap can be used for tissue interposition as an adjunct to improve healing. Various interposition flaps have been described in the literature. These include omentum, peritoneum, labial, and gracilis muscle flaps [9–11]. When operating transvaginally, the Martius flap can be used. The Martius flap is derived from the labial fat pad [11]. The flap is tunneled under the labia minora to the site of fistula repair [11]. Because of this patient's prior history of malignancy and pelvic radiation, the tissue integrity of the labia was likely compromised. Therefore, we opted to use a biologic graft made of CPT instead of a native tissue flap to provide additional support for the fistula repair. Our case is unique with the usage of CPT to augment the closure and thereby reduce the risk of fistula recurrence.

A paucity of studies exists on biologic grafts used in the setting of vesicovaginal fistulas. Caja et al. reported on the use of CPT interposition to decrease the risk of vesicovaginal fistula formation [12]. Due to the low cost, ease of handling, and less immunogenicity, CPT may be used to help prevent fistula formation [13]. Literature from colon and rectal surgery reports good success with utilizing porcine submucosa to close fistula tracts of the anus [14].

Traditionally, colpocleisis was used to treat advanced pelvic organ prolapse in patients that no longer wished to be sexually active [15]. In 1942, Latzko reported a technique (derived from Simon's procedure), whereby vault vesicovaginal fistulas were treated through a purely vaginal approach. It consists of a vault colpocleisis without any attempt at the dissection of the fistulous tract [16]. A review of literature revealed a retrospective study done that examined the success of the procedure [17]. Over a 15-year period from 1991 to 2005, the procedure was done to repair 11 VVFs. The mean size of the fistula was 12 mm and all eleven fistulas healed. No intraoperative complications occurred and only one postoperative lower urinary tract infection resulted. The mean hospital stay was 6 days. In select patients, the approach is a safe and effective option.

The radiated surrounding tissue and morbid obesity were some of the specific challenges unique to our patient. Unlike the Latzko procedure, we completely dissected the fistulous tract and sutured it closed without tension. Fully dissecting the fistula tract is somewhat controversial. Cruikshank [18] and Raz [19] reported a 100% success rate in their respective series of 11 and 65 patients without fistulous tract dissection. Because of the impaired blood supply of the surrounding irradiated tissue, we decided to fully dissect the fistula tract to optimize healing. Following the operation, the patient was discharged home on the same day and avoided any hospital stay. The major disadvantage of the technique is it has to be patient-specific as they will no longer be able to be sexually active in the future. In patients who no longer desire to be sexually active, this is a viable and safe approach to repair fistulas in challenging patients with significant medical comorbidities.

## 4. Conclusion

Vesicovaginal fistula is a rare clinical entity that is associated with high morbidity for many women. A thorough history and physical examination can help guide diagnosis, which can be confirmed using imaging such as vaginoscopy. Surgical correction is the mainstay of management and can be approached from different routes. While exposure can be more difficult, a transvaginal approach offers benefits regarding hospital stay and recovery. If the patient has additional comorbidities, as in our case with radiation and morbid obesity, wound healing appears to be an additional benefit to the transvaginal approach. Colpocleisis is a useful surgical technique to assist in closure and wound healing if the patient no longer desires to be sexually active. Such an approach provides a safe, less invasive option with the goal of decreasing morbidity.
